# Geometric Parameter Calibration for a Cable-Driven Parallel Robot Based on a Single One-Dimensional Laser Distance Sensor Measurement and Experimental Modeling

**DOI:** 10.3390/s18072392

**Published:** 2018-07-23

**Authors:** XueJun Jin, Jinwoo Jung, Seong Young Ko, Eunpyo Choi, Jong-Oh Park, Chang-Sei Kim

**Affiliations:** 1 Department of Mechanical Engineering, Chonnam National University, Gwangju 61186, Korea; harkjoon27@gmail.com (X.J.); sko@jnu.ac.kr (S.Y.K.); eunpyochoi@jnu.ac.kr (E.C.); 2 Robot Research Initiative, Chonnam National University, Gwangju 61186, Korea; jwjung@jnu.ac.kr

**Keywords:** cable-driven parallel robot, geometric parameter calibration, laser distance sensor

## Abstract

A cable-driven parallel robot has benefits of wide workspace, high payload, and high dynamic response owing to its light cable actuator utilization. For wide workspace applications, in particular, the body frame becomes large to cover the wide workspace that causes robot kinematic errors resulting from geometric uncertainty. However, appropriate sensors as well as inexpensive and easy calibration methods to measure the actual robot kinematic parameters are not currently available. Hence, we present a calibration sensor device and an auto-calibration methodology for the over-constrained cable-driven parallel robots using one-dimension laser distance sensors attached to the robot end-effector, to overcome the robot geometric uncertainty and to implement precise robot control. A novel calibration workflow with five phases—preparation, modeling, measuring, identification, and adjustment—is proposed. The proposed calibration algorithms cover the cable-driven parallel robot kinematics, as well as uncertainty modeling such as cable elongation and pulley kinematics. We performed extensive simulations and experiments to verify the performance of the suggested method using the MINI cable robot. The experimental results show that the kinematic parameters can be identified correctly with 0.92 mm accuracy, and the robot position control accuracy is increased by 58%. Finally, we verified that the developed calibration sensor devices and the calibration methodology are applicable to the massive-size cable-driven parallel robot system.

## 1. Introduction

**Figure 1:**
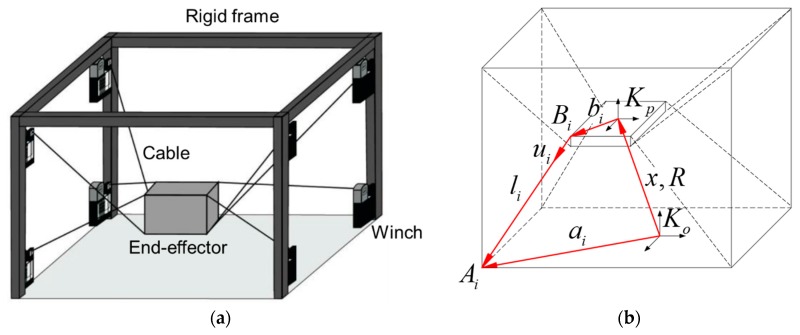
Structure schematics of a cable-driven parallel robot with eight-cable configuration; (**a**) a schematic of the eight-cable-driven parallel robot, (**b**) kinematic drawing of the cable robot.

A cable-driven parallel robot (or simply called a cable robot) is a special type of parallel robot system that is actuated by multiple cables instead of rigid links to control the robot end-effector (EE)’s six degree-of-freedom (DOF) postures. The cable-driven parallel robot is composed of four basic components: a robot EE, cables, pulleys, motor-winches, and a robot frame. The robot EE is designed with respect to the required robot task and positioned within a workspace to fulfill a specific work. The cables are connected to the EE and generate six DOF spatial motions of the EE. The motor winches control the respective cable’s length and tension for a specific task, and a rigid frame composed of steel frames and walls constitutes the robot’s workspace and maintains all the cable and EE parts inside its configuration. Figure 1 depicts the general configuration of the cable robot and its kinematic drawing.

The cable robot has advantages of large workspace and heavy payload ability in comparison with conventional parallel robots in industrial applications owing to its easy remounting, convenient transportation, and the low-inertia benefits of the actuator and EE [1,2,3]. Hence, many attempts have been executed to widen the cable robot applications such as the SkyCam [4], the Cablecam [5], a demonstrator in the German Pavilion at the EXPO 2015 [6], a high-payload transportation system [7], and a large-workspace motion simulator [8].

Despite those advantages, problems still remain in cable robots. First, more actuators than the necessary DOF movements are required to implement free spatial motions owing to the unidirectional actuation (pulling) nature of the cables. Next, a large frame is required to secure the workspace of the cable robot. The first problem causes the over-constrained robot kinematics issues discussed in [9], and the second problem causes uncertainty in the robot kinematics parameters. Although the cable robot can be installed easily and possesses a variable configuration by relocating the position of the pulleys, calibration must be performed to achieve a high accuracy control; however, the aforementioned problems render challenges in attaining a qualified calibration. In detail, the kinematic equation of the eight-cable driven parallel robot shown in Figure 1b is given by $l_{i}=a_{i}-x-Rb_{i}$ [7]. The geometry of the robot is described by its proximal anchor points on the robot base $A_{i}$, and the distal anchor point on the EE, $B_{i}$, which are defined by the vectors $a_{i}$ and $b_{i}$, respectively. The index *i* denotes the cable number, and *m* is the absolute number of cables. Further, x and R describe the position and orientation, respectively, of the EE fixed frame $K_{p}$, with respect to the world coordinate frame $K_{o}$. Regarding the kinematic equation solver and control algorithm development, it is clear that all the configuration parameters need to be accurately determined before implementation, especially $a_{i}$ for the large-size cable robot development.

Generally, to calibrate the kinematic model, the model must include the most significant geometric and nongeometric parameters that influence the control accuracy. For the cable robot, three types of geometric errors exist. The first type is a machining error of winches such as the drum diameter or the pitch length. The second is EE’s geometric parameters. The third is the position errors of the EE’s cable connection points and the pulley where the cable comes out [10]. Nongeometric errors include cable elongations or the cable sagging effect caused by gravity and the cable material property.

Parallel robotic calibration methods [11,12] have been actively researched for decades with some promising results. However, cable robot calibration is an open issue. Tadokoro et al. reported a calibration method for a portable rescue crane where a mobile cable robot used for the calibration is typical [13]. Borgstorm et al. proposed a self-calibration method based on the position and forces differences of a planar two-transform robot with four cables [14]. The error of the calibrated robot is determined as 19.8 mm for the tension-based self-calibration, and 6.3 mm for the position-based calibration. Miermeister et al. developed a calibration method through differential kinematics model of cable robots and applied both conventional as well as auto-calibration methods to spatial cables with eight cables [15,16]. As the behavior of the cable robots does not depend only on the geometric parameters but notably on the material parameters such as the cable stiffness and mass, the method uses a multistage parameter identification [17]. Generally, because a force sensor contains noise and draft errors, a limit exists in performing a precise calibration; therefore, it is difficult to perform a high-precision calibration. Sandretto et al. proposed the calibration of the RellAx8 robot using weighted least-square and self-calibration, where the model of the robot consists of the proximal and distal anchor points as well as the offsets of the cable length. However, the efficient calibration could be performed only with expensive measurement devices [18], and the most representative sensor is the laser, LaserTraker [19]. For the commercialization of cable robots, cost-efficient calibrations [20] including auto-calibration are required.

Auto-calibration or self-calibration methods rely on the measurements of the internal sensors of the robots. In this case, it is required that the *n*-DOFs robot contains *m* > *n* internal sensors. However, no specified sensors are available hitherto for the convenient and inexpensive calibration that can be performed in a short time. Further, most of the previous studies focused on the EE posture calibration based on the lumped uncertain kinematic parameters that may cause different kinematic calibration parameters for the different postures or motions of the robot. Further, the cables provide only unidirectional actuation as they can only transmit tension force, not compression force; thus, the cable elongation must be considered for the robot calibration. Among some kinematic uncertainties contained in the cable robot, the position error of the EE’s cable connection points can be minimized by machining the EE with a three-dimensional (3D) printer [20] or by measuring the accurate 3D shape measurement device before installation, as the rigid frame is small and the pulley position can be easily measured offline. However, if we consider a wide cable robot, the calibration of the pulley’s position, which is essential in the cable robot kinematics, is another challenge for the precise robot motion because it is difficult to measure the pulley position directly within the wide-structured robot frame.

We present herein a novel cable-driven parallel robot calibration methodology based on a newly prototyped calibration sensor device made of commercially available laser distance sensors. The methods also utilize the necessary cable robot modeling such as cable deformation, pulley position identification, and robot kinematic models for accurate robot calibration. In terms of those system modeling, identification, and calibration methodology implementation to over-constrained cable robot in 3D space, this paper is a significant improvement over our previous calibration method of the cable robot in 2D plane workspace [20,21].

The laser distance sensor is a cheaper and easier option to incorporate than the laser tracker that is typically used in previous calibration methods. Further, the laser distance sensor has a larger measuring range than the IR sensor; subsequently, it can be applied to a cable robot calibration with a wide working range. Because the proposed method uses the laser distance sensor, it is equivalent to a type of an external calibration method. However, the proposed methodology and algorithm encounters the cable models and the kinematic constraints, and the inexpensive calibration sensor could yield accurate calibration results with few measurement points in the Cartesian space. Moreover, the algorithm is simpler and easier to implement than the existing self-calibrating method [14] or image processing [22].

This paper is organized as follows: in Section 2, we present the overview of the over-constrained cable robot and the suggested calibration methodology. This is followed by the detailed explanation of the first-stage pre-calibration of the equipped components of the cable robot in Section 3. In Section 4, polymer cable modeling and extended pulley kinematics to improve the calibration accuracy will be discussed. Online calibration procedures including sensor measurement and kinematic parameter estimation are presented in Section 5. The experimental setup and results are shown in Section 6. Our concluding remarks and future works are presented in Section 7.

## 2. Overview of the Cable-Driven Parallel Robot and the Calibration Methodology

### 2.1. The MINI Cable Robot

**Figure 2:**
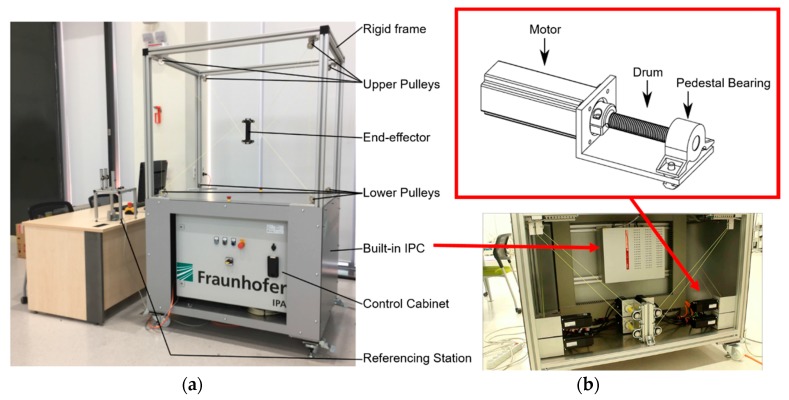
The MINI cable robot; (**a**) a photo of the whole MINI cable robot system and (**b**) a winch-motor drawing and actual installation.

The MINI cable robot hardware and controller structures in this study are shown in Figure 2. The primary hardware components of the cable robot are an aluminum robot frame, motor winches, pulleys, a built-in industrial personal computer, and a control panel equipped with motor drivers and power supply. The cable robot was built by Fraunhofer IPA (Stuttgart, Germany) in collaboration with Robot Research Initiative (Gwangju, South Korea) as a cable robot research platform to develop and test the hardware, control algorithm, calibration method, architecture designs, etc., before proceeding to the actual system application. The cable robot is a type of fully driven parallel robot with eight cables and the respective eight motor-winch system that winds and unwinds each polymer fiber cable to control the six DOF motion and posture of the EE. The cable robot is an open-architecture and modular robot that can be displaced within the laboratory and operated by any Windows PC.

**Figure 3:**
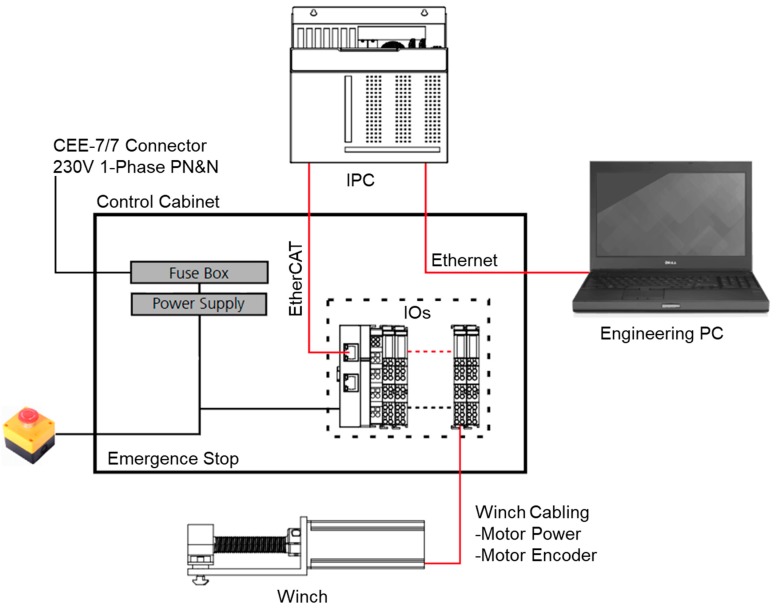
MINI cable robot controller schematics.

TwinCAT3 is used for the real-time control software of the cable robot. The software is used as a programmable logic controller (PLC) and a time controller that supports the CNC system for the kinematic transformation and motor control. In detail, the TwinCAT3 consists of a real-time controller TwinCAT XAE based on Microsoft Visual Studio 2013, and a human-machine interface (HMI) TcHMIPro. The TwinCAT XAE is a controller package that allows additional programmability and control algorithm implementation over the PLC. The PLC assists the driving and monitoring of the robot system and acknowledges the CNC system in responding to a certain request. It also provides an interactive platform for implementing different user algorithms and an external commendation to the process outputs. The data in all the modules of the CNC channel can be accessed over the high-level interface (HLI). The HLI is a shared memory area created for access by both the CNC and PLC to establish data exchange between the CNC and PLC. In addition to the real-time components, TwinCAT3 also provides the HMI. This exemplary interface provides an “end user” ability for high-level interactions with an operator, such as programming robot trajectories and running predefined commands. This interface does not have real-time implementation capability, but it allows an easy interaction between the robot and the operator or another robot. This interaction is freely programmable. The interaction between the different hardware components occupies the CANopen field bus. The CANopen field bus carries commanded position values to the servo amplifiers and digital force values to the control PC. The control cabinet is shown in Figure 3. 

In this study, we used the aforementioned MINI cable robot for experiments and model derivation to verify the performance of the suggested calibration algorithm. As the cable robot configuration is the same as the industrial eight-cable robot system, we can easily transform the developed algorithm to the different-sized cable-driven parallel robot from the results of this study onto the MINI cable robot.

### 2.2. Cable Robot Calibration Procedures

The suggested auto-calibration methodology comprised five stages as follows: a pre-calibration stage for the parts calibration equipped in the cable robot, a modeling stage, a measuring stage, an identification stage, and an adjustment stage. At the pre-calibration stage, eight motor winches to the cable length and force sensors are calibrated under the assumption of no deflection in the cables, to increase the accuracy of the cable robot. As the motor-winch and cable length are the primary actuators of the cable robot system, the ideal cable length must be obtained from the amount of winch rotational angle for the robot kinematics computation. Further, the force sensor to measure the cable tension is necessary for the force control of the cable robot and must be calibrated before the actual operation. A modeling stage is incorporated to compensate the effect of cable deformation caused by the cable length, payload, and pulley geometry during the calibration. As the utilized cable is made of a polymer material, the cable elongation is attributed to the error in the robot motion and must be considered. The pulley geometry also affects the cable length error from the wrapping lengths around the pulley. The results of the first two stages are utilized in the last three stages of the calibration processes. We utilized mathematical models derived from the first two stages to compensate those negative effects to the cable robot. The remaining measuring, identification and adjustment stages are the actual procedures of the robot calibration for the accurate EE posture control by determining the cable robot kinematic configuration parameters including the pulley position in the frame.

Once the structural parameters of the cable robot configurations are determined, the whole process of robot calibration is performed automatically by utilizing the laser distance sensor. In other words, by using the measurement of the EE position from the laser distance sensor, we can identify the uncertain parameters of the cable robot kinematics.

## 3. Pre-Calibration of the Parts Equipped in the Cable-Driven Parallel Robot

This step must be performed before executing the auto-calibration algorithm of the cable robot. The winch calibration and force sensor calibration consist of two processes, and a hardware device of home-position reference platform.

### 3.1. Motor-Winch Calibration

**Figure 4:**
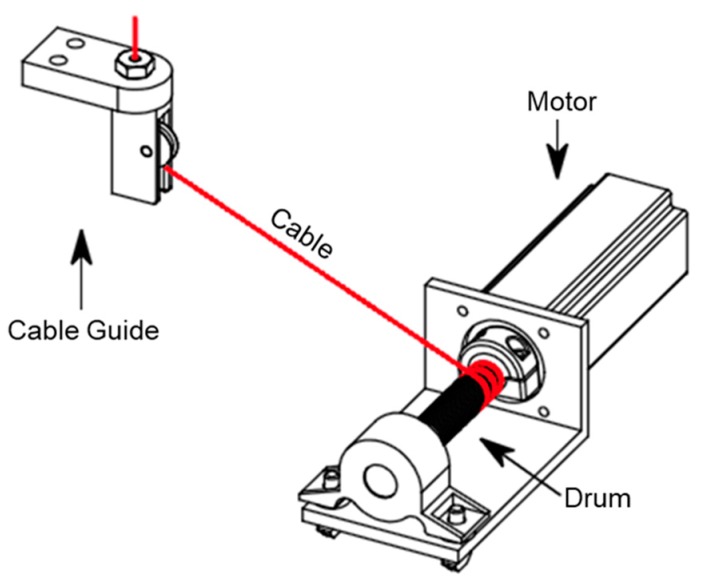
A schematics of the motor-winch component and course of the cable with cable guide pulley.

The motor-winch is a key component of the cable robot that yields the motion of the EE through the cable length and tension. Two mechanical components were used as shown in Figure 4; the first component is the cable drum that converts the rotational angle into lateral cable displacements, and the second component is the pulley. The pulleys are used to guide the cables in and out of the winch to connect onto the EE.

The transmission ratio has to be defined for each winch that describes the relationship between the drum rotating angles measured by an encoder and the cable lengths. It depends primarily on the ratio of the gearbox and the circumference of the drum. However, it is also influenced by the guiding unit, cable diameter, and cable tension owing to the flattening effects. By assuming the ideal cable case in which the cable properties are uniform, we can derive the cable length from the motor winch as the following:

(1) $$L_{i}=\sqrt{{\left(\pi d\right)}^{2}+p^{2}}\cdot \theta_{i}\;$$

where $L_{i}$ is the winding and unwinding cable length at each winch’s outlet, i=1,...,8 is the respective number of cable, d is the diameter of the drum, p is the drum pitch, and $\theta_{i}$ is the rotational angle of each drum. In Equation (1), the calibration results show that the cable length error between the actual measurement and the calculated values is less than 0.1 mm, under the no-payload condition.

### 3.2. Force Sensor Calibration

**Figure 5:**
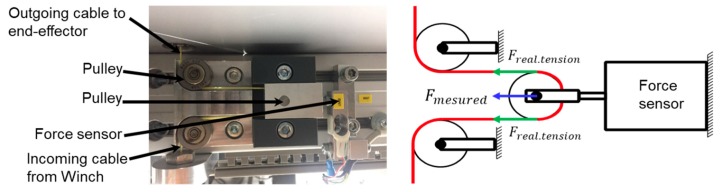
Principles for cable force measurement.

Eight force sensors are attached in front of each motor winch with a special mechanism to measure the cable tension through the force sensor for the further improved calibration performance and control performance, as shown in Figure 5.

**Figure 6:**
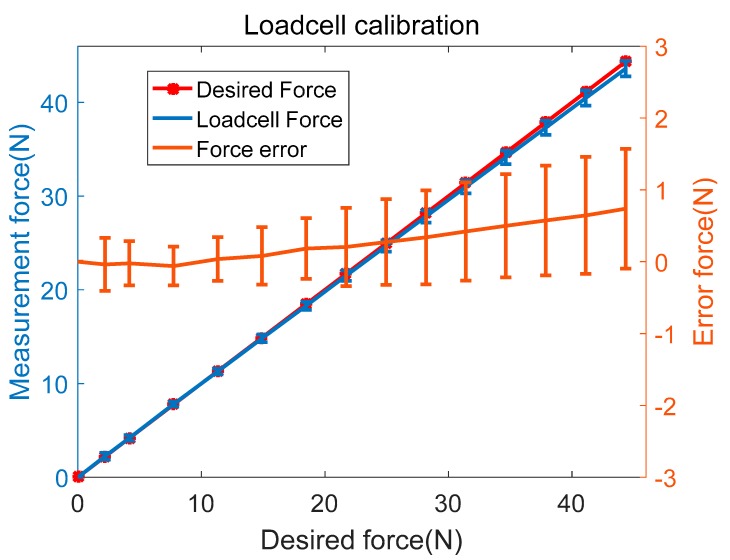
Force sensor calibration results.

The advantages of this mechanism are that the force sensor can be covered and protected by the housing and the electric wiring can be fixed inside the robot frame. From the sensor specifications and wiring mechanism of the pulley in Figure 5, the actual tension of the cable $F_{Tension}$ can be obtained from the force sensor measurement, $F_{Measured}$, as $F_{Measured}=2\cdot F_{Tension}$. This equation is derived by known mass experiments on this force measurement mechanism that could be verified by experimental results, as shown in Figure 6, where the yellow line (ideal) is the known cable force, and the orange line (cable7) is the actual force sensor measurement. The difference between the two values (green line) shows that the force sensor calibration error is smaller than 1 N, and is an acceptable value for the cable robot.

### 3.3. Home Position Reference Platform

**Figure 7:**
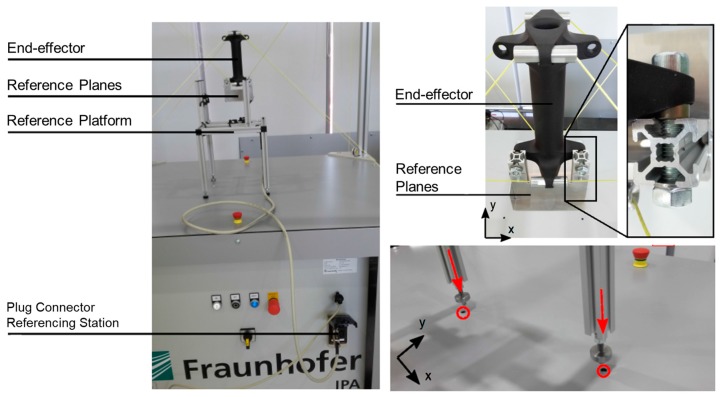
Complete hardware setup to start the referencing procedure.

**Figure 8:**
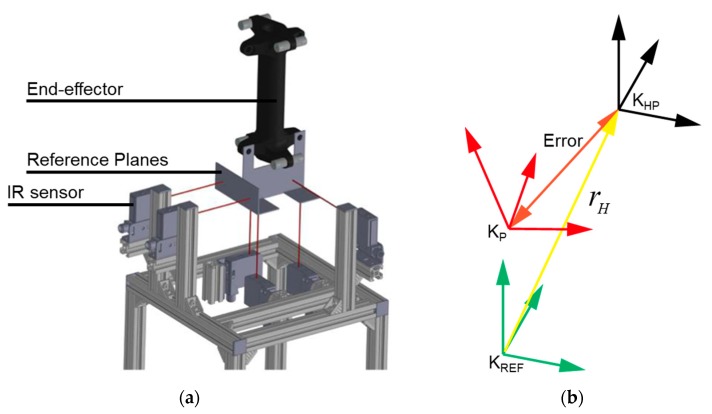
Reference platform and coordinate system for the home position reference platform; (**a**) attachment of the reference planes at the platform, and (**b**) reference platform coordinate.

All the geometric parameters of the cable robot are defined with respect to the base coordinate in the Cartesian space. Subsequently, a device is required that can precisely set the home position in the space. Hence, we designed and fabricated a home-position reference platform, as shown in Figure 7. The home-position reference platform is made by aluminum profiles and six IR sensors (GP2Y0A41SK0F, SHARP, Hsinchu, Taiwan) to place the EE at the same position inside the cable robot workspace during calibration. The coordinate system shown in Figure 8 is defined by the following; $K_{ref}$ is a coordinate axis of the reference platform, $K_{HP}$ is a coordinate axis of the home position, and $K_{p}$ is a coordinate axis of the EE. As the home-position reference platform is to adjust the position and posture of the robot EE at the origin, therefore the position repeatability error is zero.

## 4. Models to Compensate Uncertainties of the Cable-Driven Parallel Robot

### 4.1. Polymer Cable Modeling

The cable is a crucial part of the cable robot system and its characteristics significantly influence the robot performances [7]. In particular, a standard mathematical model of the cable behavior does not exist that compensates the cable uncertainties including elongation, degradation, creep, and so on. In this study, we assume that the cable behavior makes nongeometric errors [23], and thus, we conducted the cable deformation experiments and derived the surface fitting models of the cable elongation in terms of the cable length and tensions. From the MATLAB surface fitting results, we obtained the fifth-order polynomial equation as the following:

(2) ΔL =f(cable_length, cable_tension) 

**Figure 9:**
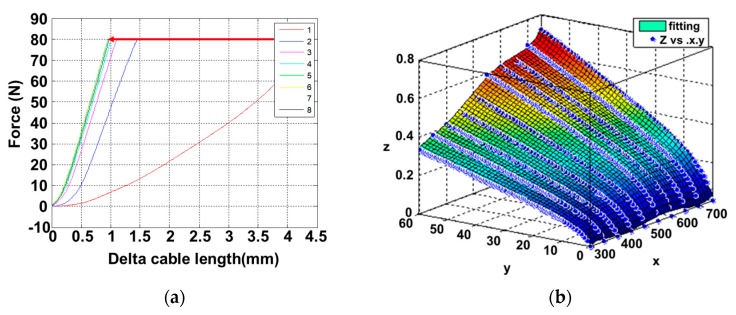
Polynomial surface model of the cable elongation as a function of cable length and cable tension measurement; (**a**) experimental data for different cable lengths and tensions, and (**b**) surface-fitted model where x is the cable length, y is the cable tension and z is the cable deformation.

Figure 9a shows the cable model experimental data, and Figure 9b shows the surface-fitted function from the experimental data. The fitted cable elongation model obtained by the known tension and cable length to the polymer cable deformation is used to estimate the cable length error during the calibration process, and to ensure that the robot operates properly. The experimental results validate that the surface-fitted polynomial equation model can estimate the cable deformation with relatively small error.

### 4.2. Pulley Kinematics

**Figure 10:**
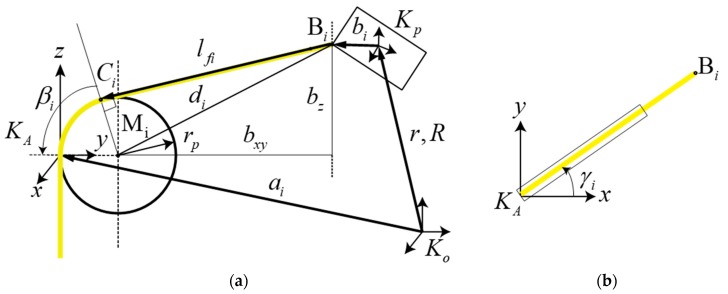
Extended pulley kinematics to encounter the pulley geometry into the cable robot kinematics; (**a**) determination of the point $C_{i}$ where the cable exit, and (**b**) pulley rotation angle $\gamma_{i}$ out of the xz -plane.

Several pulleys are equipped with the cable robot to guide the cable from the motor winch to the EE. However, owing to the cable exit point varying according to the EE motion, the cable length error caused by the pulley geometry must be considered [10,24]. To derive the pulley geometry influence on the cable length, a new coordinate system is introduced to define the pulley orientation. Based on this coordinate frame, a pulley kinematic model is defined to obtain the panning angle for the pulley, and the resting angle of the cable wrapped around the pulley. The exit point is affected by both angles. The resting angles define the length of the cable on the pulley, to add to the free spanning length from the pulley to the EE. These angles are dependent on each other; subsequently, they can be calculated by the pulley kinematic equations. The extended kinematic equation considers the pulley mechanism illustrated in Figure 10, in which the length of the *i*-th cable is obtained by:

(3) $$l_{i}=l_{fi}+\theta_{i}r_{p}\;$$

where $l_{fi}$ is the actual cable length used for the cable robot kinematic equations obtained by considering the pulleys geometry:

(4) $$l_{fi}=c_{i}(r,R)-r-Rb_{i},\;c_{i}=a_{i}+R_{A}(R_{z}(\gamma )((I-R_{y}(\beta )){\left[r_{p},0,0\right]}^{T})),\mathrm{and}\;\theta_{i}=\arccos(\frac{\sqrt{d_{i}{}^{2}-r_{p}{}^{2}}}{\sqrt{{\left(d_{xy}-r_{p}\right)}^{2}+b_{z}{}^{2}}})+\arccos(\frac{b_{z}}{\sqrt{{\left(d_{xy}-r_{p}\right)}^{2}+b_{z}{}^{2}}})\;$$

The required values and geometrical parameters are described in Figure 10. As the pulley is relatively small and can be produced the same size as the drawing, the manufacturing errors can be ignored.

## 5. Measurement, Identification, and Adjustment Process

### 5.1. Measuring Stage

One could expect that the final error of the calibration procedure generally decreases when incorporating numerous calibration data in different poses because the influence of statistical measurement errors and noise is canceled when computing with higher numbers of measurements. This intuition from conventional physical measurements is not always true because a large number of experimental measurements used in the parameter fitting may result in negative influences on the numerical computation and conditioning of the fitting algorithm. The numerical errors caused by larger test sets can exceed the benefits owing to the statistical effects. Therefore, one searches the best calibration measurement by tradeoff; either a high number of pose data for averaging the measurement errors, or a low number of poses for small numerical errors.

The effectiveness of the whole calibration procedure can only be assessed by experimental verification. In contrast, one can improve the numerical conditioning of the problem by the proper selection of the poses for the measurement posture sets. This selection can be supported by numerical simulation. Furthermore, one has to consider that the improvements through calibration are achieved in a Section of the workspace around the measurement posture sets. As observed in other works [15], a minimum number of cable robot calibration sets exists for the condition number of approximately 25 poses. Hence, in this study, we measure 25 different posture data and utilized them in the cable robot calibration.

**Figure 11:**
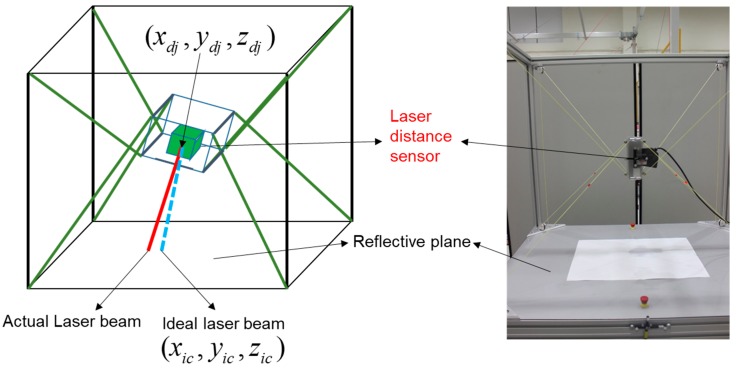
Laser distance sensor setup.

As shown in Figure 11, the laser distance sensor is mounted at the center of the robot’s EE. Further, at each different 25 postures, we measure the laser distance sensor data and utilize them for the next processes of the cable robot geometry calibrations. The sensor beam position difference between the ideal laser beam distance obtained by kinematic equations and the actually measured laser beam distance indicates kinematic parameter errors. Ultimately the laser beam position difference necessitates the robot calibration. Briefly, the proposed calibration method is derived to match the ideal laser beam distance and the actual laser beam distance.

**Figure 12:**
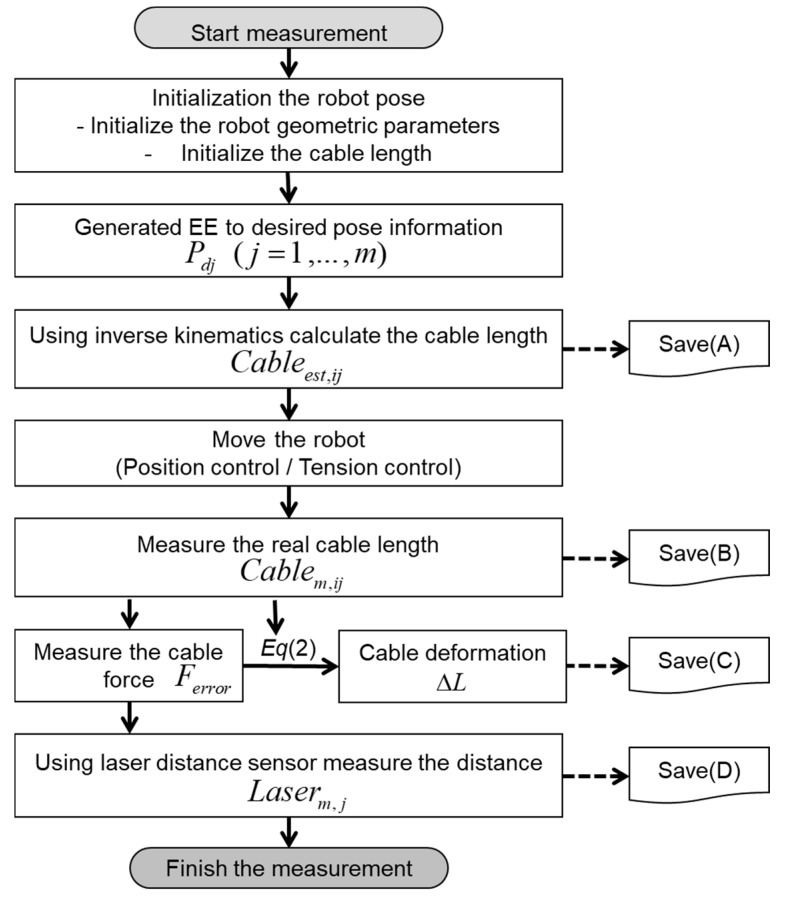
Data acquisition phase.

The measuring stage is depicted as a flowchart, as shown in Figure 12, where we acquired the necessary values by moving the EE of the cable robot. First, we define the desired positions $P_{d\text{​}j}$, and calculate the desired cable length $Cable_{est,jj}$, through the inverse kinematics of the cable robot.

For the desired position, we save the Cartesian position and cable length as [Save (A)] in the flowchart of Figure 12. Without the robot calibration, owing to uncertain kinematic parameters including the incorrect pulley position of the large robot frame size, inaccurate motion resulted and the EE moves to another position that is defined by $P_{j}$. Next, we move the EE of the robot to the previous desired position using the position control. As the length of each cable obtained from the uncalibrated kinematics contains uncertainty caused by the inaccurate pulley position information and the uncalibrated kinematic parameters, some cables result in a large tension or sagging. Here, we measure and store the actual cable length $Cable_{m,jj}$, as described by [Save (B)] in the flowchart. Further, we measure the cable tension $F_{measured}$, as in [Save (C)] of all the cables. In this posture, the laser distance sensor measurements are also saved as a value of $Laser_{m,j}$ in the [Save (D)] steps in Figure 12, where *i* is the number of cable and *j* is the number of measurement pose defined. Finally, we could prepare all the information for the kinematics calibration in the next step.

### 5.2. Identification Stage

**Figure 13:**
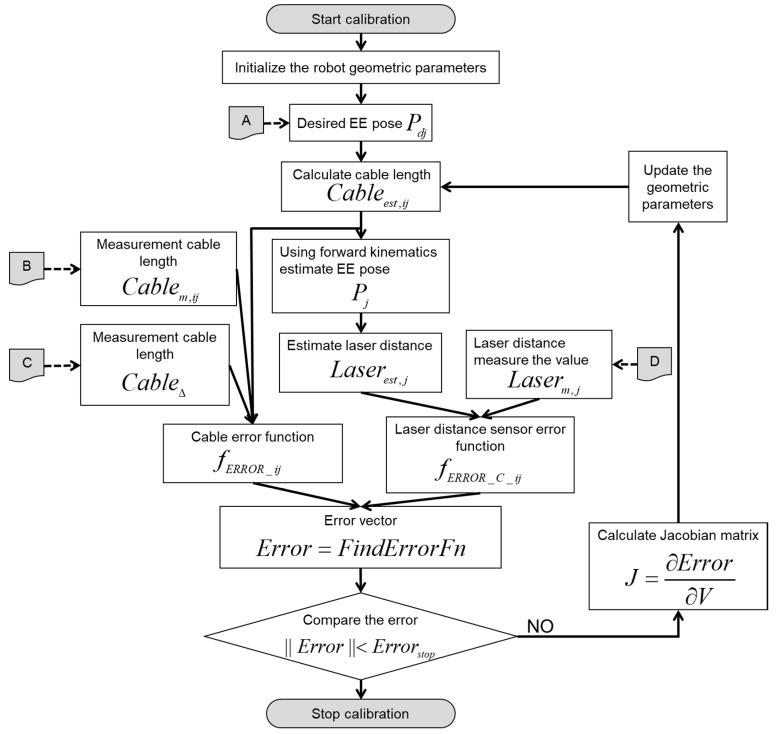
Flowchart of parameter estimation using optimization method in the identification stage.

The parameter identification procedure using optimization with the iterative searching method is summarized in Figure 13.

To obtain the calibrated robot kinematics under the assumption above, we define the geometric parameter vector, $V=\left[x_{2},\;x_{3},\;x_{4},\;x_{5},\;x_{6},\;x_{7},\;y_{2},\;y_{3},\;y_{4},\;y_{5},\;y_{6},\;y_{7},\;z_{5},\;z_{6},\;z_{7},\;z_{8}\right]$, of the cable robot having 16 parameter elements, where the elements of the geometric parameter vector are the respective cable length to be estimated. As the calibration initially starts from the same EE position and the cable robot’s plane is constant after the first leveling, we can reduce the number of unknown geometry parameters to 16 from a total of 24 parameters (3 coordinate values times 8 cables). To obtain a solution for the problem, as previously mentioned, we utilized the measured and saved data sets; the difference between the measured laser distance ($Laser_{m,j}$) and the predicted laser distance ($Laser_{est,j}$), and the difference between the measured cable length ($Cable_{m,ij}$) and the predicted cable length ($Cable_{est,ij}$).

As we know the measured cable length, $Cable_{m,ij}$ to predict $P_{j}$, we can perform forward kinematics [20] using the current $a_{i}$ value. Forward kinematics obtains the pose of the EE after performing an iterative calculation to minimize the length difference in the vector closed loop obtained from the inverse kinematics. Equation (3) can be transformed into a cable length error function formulated by Equation (5) using the given current pulley positions $a_{i}$, and the measured cable lengths $Cable_{m,ij}$:

(5) $$f_{ERROR}{}_{\_ij}=(c_{i}(r,R)-r-Rb_{i}+\theta_{i}r_{p})-Cable_{m,ij}\;$$

To solve the forward kinematics in the given equation, a general Newton–Raphson iterative method is used. However, when a numerical method is applied when an error exists in the frame shape, the solution does not converge and leaves a certain amount of error. The proposed calibration algorithm optimizes the geometric parameters to reduce these error values. The predicted cable length $Cable_{est,ij}$ can be expressed as Equation (6), such that Equation (5) can be modified to Equation (7); therefore, the difference between the predicted cable length and the measured cable length becomes the same as the results of $f_{ERROR}{}_{\_C\_ij}$:

(6) $$Cable_{est,ij}=c_{i}(r,R)-r_{d\text{​}j}-R_{d\text{​}j}b_{i}+\theta_{i}r_{p}\;$$

(7) $$f_{ERROR}{}_{\_C\_ij}=Cable_{est,ij}-Cable_{m,ij}\;$$

The estimated distance from the laser distance sensor is obtained by the predicted position $P_{j}$ of the EE through the cable robot’s forward kinematics. Assuming that the laser reflector is placed on a plane, the expected distance that the laser sensor can measure can be calculated. If we define the intersection of the laser beam and laser reflector point as $IC=[x_{ic},y_{ic},z_{ic}]$ the estimated laser distance can be computed by:

(8) $$Laser_{est,ij}=\sqrt{{\left(x_{ic}-x_{j}\right)}^{2}+{\left(y_{ic}-y_{j}\right)}^{2}+{\left(z_{ic}-z_{j}\right)}^{2}}.$$

As we know the actual distance from the laser distance sensor measurement, $Laser_{m,j}$, conducted in the previous step, we can easily compute the distance error function as the following:

(9) $$f_{ERROR\_L\_ij}=Laser_{est,ij}-Laser_{m,ij}\;$$

By accumulating the errors calculated using the multiple desired positions ($P_{d\text{​}j}$), a final error function can be obtained as Equation (10). The error function contains M cable errors and one laser distance error for one location. If the number of desired positions is N, subsequently, a total of (M + 1) × N error values will be obtained. Hence, the error vector size becomes ((M + 1) × N) × 1:

(10) $$\begin{array}{l} Error=FindErrorFn(V,b_{i},Cable_{m,ij},Laser_{m,j},P_{d\;j})=\\ {\left[\begin{array}{ccccccccc} f_{ERROR\_C\_11} & \cdots & f_{ERROR\_C\_M1} & f_{ERROR\_L\_1} & \cdots & f_{ERROR\_C\_1N} & \cdots & f_{ERROR\_C\_MN} & f_{ERROR\_L\_N} \end{array}\right]}^{T} \end{array}\;$$

where the geometric parameter, V, is a variable that expresses the calibrated pulley position $a_{i}$_,_ which are independent variables to be calibrated. In the error function, *Error* is a nonlinear function involving the numerical iteration for forward kinematics, and the best-case result is Error = 0. In other words, V is the solution of the error function in Equation (10). We use the numeric iterative method to obtain the *Error* function’s solution. Hence, we calculate the discrete Jacobian matrix *J* as the following Equation (11), and the (K + 1)-th geometric parameter sets in the iterations computation is obtained using the K-th V and *J* as shown in Equation (12):

(11) $$J=\frac{\partial Error}{\partial V}=[\begin{array}{ccc} \frac{Error(V_{1}+\Delta J_{1},\cdots ,V_{K}-Error(V_{1},\cdots ,V_{K})}{\Delta V_{1}} & \cdots & \frac{Error(V_{1},\cdots ,V_{K}+\Delta J_{K})-Error(V_{1},\cdots ,V_{K})}{\Delta V_{K}} \end{array}]\;$$

(12) $$V(K+1)=V(K)-\lambda J^{+}Error\;$$

where $J^{+}$ represents the pseudo-inverse of the Jacobian, and *λ* (0 < *λ* < 1) represents the under-relaxation parameter for convergence safety. The iteration computation is continued until the absolute value of *Error* becomes less than the preset Estop ($\left\|Error\right\|\leq E_{stop}$), and the value of the ending point becomes the final geometric parameter vector $V_{final}$.

### 5.3. Calibrated Parameter Adjustment Stage

We utilize the automation device specification (ADS) of the controller to implement the online calibration procedures. The ADS is the communication protocol of TwinCAT. It enables the data exchange between two different devices. The ADS is media-independent and can communicate via serial or network connections, and the necessary hardware interfaces are provided for communication with third-party software such as MATLAB. We programmed the identification algorithm in MATLAB and implemented the online auto-calibration method using ADS in real-time communication with a PLC controller for the robot motion control. Finally, we can obtain the real-time geometry parameter through MATLAB and adjust the calibrated pulley geometric parameters to the actual on-site controller.

## 6. Experimental Validation of the Auto-Calibration Method

**Figure 14:**
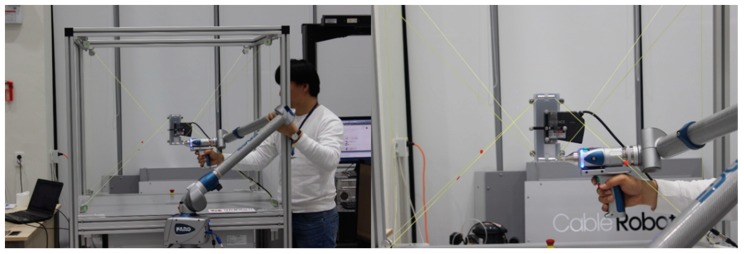
A photo-shoot of the actual measurements of the end-effector position using Faro edge ScanArm equipment.

We conducted the cable robot EE control experiments for different pulley position information by referencing ISO9283 standards that describe robot accuracy measurements. The performance of the suggested method can be verified by comparing the actual EE’s position and the posture accuracy comparison for the different values of $a_{i}$. As show in Figure 14, the EE’s six DOF position and postures are measured by a Faro edge ScanArm instrument. The MINI cable robot was used for the experiments and the explained algorithms were implemented on the real robot controller for the actual validation. To assume the unknown frame size of the MINI cable robot, the initial guessed frame size was set by the 3D Solidworks drawings, and the experiment was conducted with no warm up before the calibration. To verify the performance of the suggested methodology, we tested the algorithm for the three different values of $a_{i}$; (1) drawings from the 3D modeling tool in SolidWorks, (2) 3Dl position measuring Equipment (Faro edge ScanArm HD) and (3) calibration data from our algorithm. The measured data are shown in Table 1.

**Figure 15:**
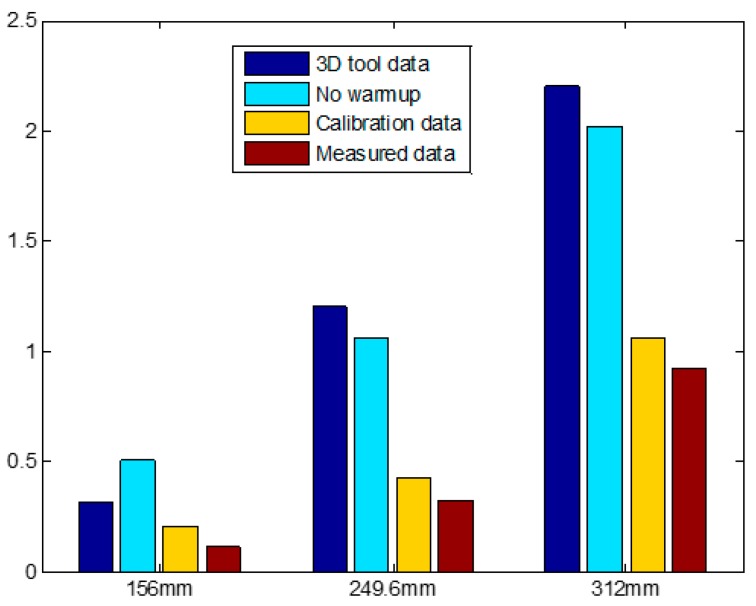
Experimental results.

Figure 15 shows the summarized results of the geometric error obtained by comparison between three different geometry identification methods in Table 1, and the actual robot size measurements. For the MINI cable robot, the center of the workspace and thus the measurement box [25] coincide with the origin of the coordinate system. Hence, the 100%, 80%, and 50% box sizes are 312 mm, 249.6 mm, and 156 mm, respectively. In the results, the measurements from the precise 3D reverse drawing equipment show the best performance, and the proposed single-laser distance-sensor-based method shows the second most accurate results. However, the geometry error differences are insignificant (under 0.2 mm in each coordinate); subsequently, we verified that the proposed method can be used as a high-quality geometry calibration method with inexpensive sensor utilization.

**Figure 16:**
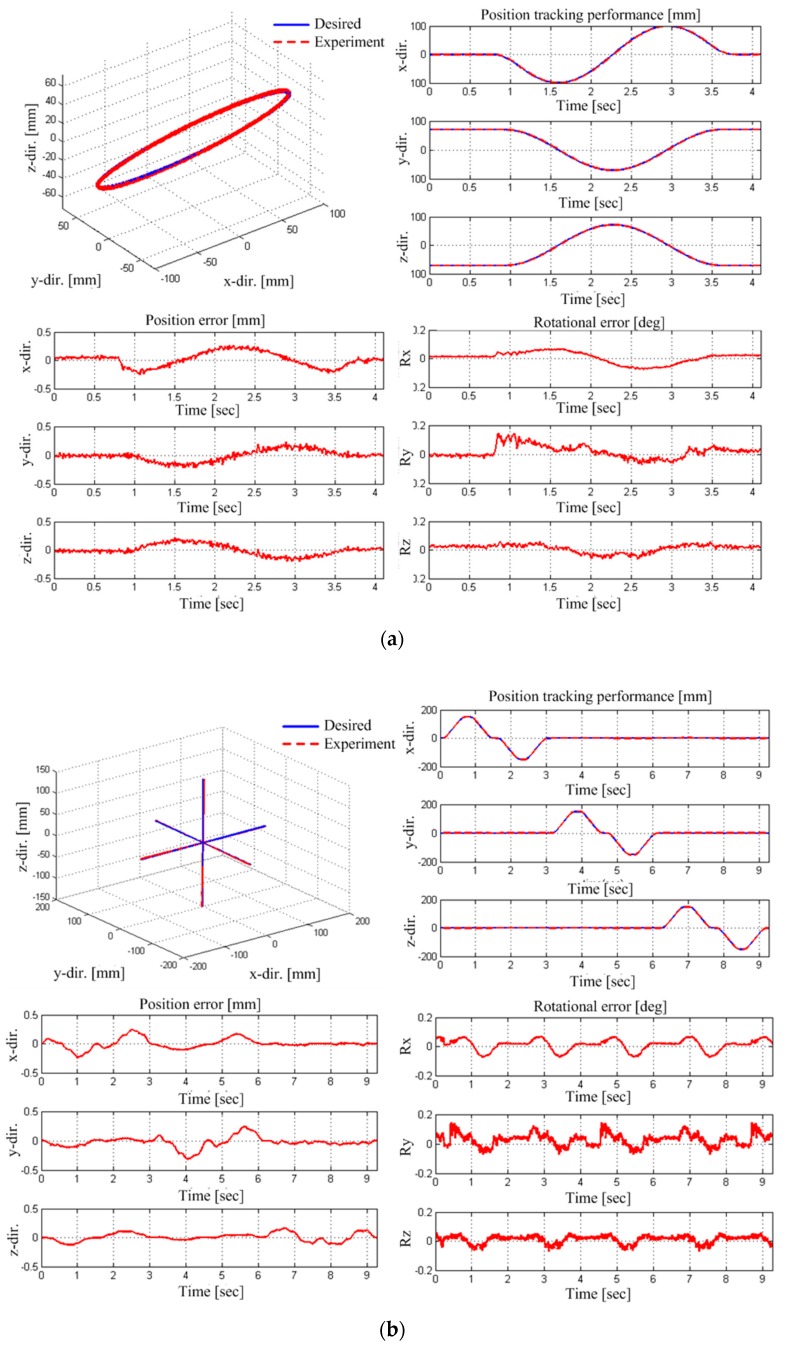
Path accuracy experimental results; (**a**) circular trajectory and (**b**) linear trajectory.

Figure 16 depicts the path accuracy results from the EE motion control; for a circular movement in (a) and for a linear movement in (b). The rotational angles references were set to zero to focus on the EE position control performance in both experiments. As results, the path accuracy is 1.284 mm and the path repeatability is 0.18 mm after calibration.

Although significant efforts are undertaken in the design, manufacturing, and assembly of the robots to build the robot accurately according to the design specifications, errors and uncertainties in the geometry of the robot are inevitable. Using costly procedures, the errors can be reduced but not eliminated. While machine parts such as drums, pulleys, and guidance systems can be manufactured with narrow tolerances by virtue of manufacturing technology advances, the setup of large machine frames still suffers from assembling errors. Thus, the improvement in the accuracy by calibration during the initial operation of the robot must be addressed. A detailed technical definition of the robot accuracy and measuring process are subject to ISO 9283, where a calibration is defined as the procedure to estimate the actual numerical values of the geometrical design parameters of the robot; the accuracy is shown as a key performance indicator for all kinds of robotic devices, and the position accuracy of a robot describes its ability to move its reference points to the desired absolute position in space. Regarding this, the experimental results show that the proposed calibration method could be a novel online geometry error compensation method to satisfy the industrial cable robot’s requirements and to improve the accuracy of the cable robot system.

## 7. Discussions and Conclusions

We present herein a geometry calibration methodology for a cable-driven parallel robot system utilizing a single commercially available laser distance sensor. To improve the accuracy of the calibration against structured and unstructured kinematic uncertainty, a polynomial polymer cable model, an extended pulley kinematics, and a cable robot kinematics were incorporated. Further, we suggested step-by-step procedures of the cable robot calibrations comprising five stages from the pre-calibration of the structured component in the cable robot system to the auto-calibration method for practical implementation in the real world.

The suggested methodology could overcome the uncertain kinematic parameters of the cable robot such as cable elongation and pulley inclusion error, and estimate the cable robot configuration by identifying the pulley position on the cable robot frame. The simulation and experimental results conducted on the MINI cable robot system could verify the performance of the proposed calibration methods. Considering the cable robot application with a large workspace and a frame size, the suggested method has the potential to overcome the unstructured and structured uncertainties in the cable robot kinematics, especially for spatial pulley position estimation that is essential for computing the forward and inverse kinematics of the cable robot.

Compared to the previous cable robot calibration method that incorporates multimodal measurements from expensive sensors, we could achieve inexpensive and convenient but accurate methods to calibrate the cable robot by a model-based approach composed of kinematic parameters and experimental-data-driven mathematical models. In this study, we proved that even with insufficient sensor measurements for the cable robot calibration, the mathematical modeling of the unknown parts and the experimental data could overcome the insufficient sensor measurements. Moreover, the error modeling of the sensors will improve the accuracy of the cable robot calibration [25]. In comparison with the vision camera and image processing based calibration methods, the suggested method has benefits of inexpensive cost, applicability to a large size cable robot system, and relatively ease calibration procedure with real-time computation. However, since the calibration result depends on the laser distance sensor performance and mathematic models derived from the experiments, the robustness problem comes from un-modeled elements may arise during the long-term operation. The proposed methodology has a potential to be applicable to a different configuration cable robot, but the kinematics and the dynamic force distribution equation needs to be appropriately modified to the respective cable robot system.

In our future works, more sophisticated modeling of the uncertain components of the cable robot system, such as the strict cable deformation and elongation modeling and the pulley friction and dynamic effects on the cable, will be studied to improve the robot EE position and posture control performance as well as long-term calibration free operating. Also, the quantification analysis and sensitivity analysis between the laser distance sensor accuracy and the cable robot calibration performance will be accomplished to provide a sensor selection guideline for the real-world application of the proposed methods.

## Figures and Tables

**Table 1:** Three different pulleys geometric of $a_{i}$ (mm).

Pulley Geometric, $a_{i}$	3D Tool Solidworks Data	3D Equipment Measured Data	Calibration Data
cable #1	[500, −378, −441.5]	[506.40, −373.00, −453.20]	[506.40, −373.00, −453.20]
cable #2	[−500, −378, −441.5]	[−488.87, −374.26, −453.20]	[−490.96, −373.77, −453.20]
cable #3	[−500, 378, −441.5]	[−491.89, 381.46, −453.20]	[−494.02, 382.20, −453.20]
cable #4	[500, 378, −441.5]	[503.63, 382.76, −453.20]	[502.48, 383.00, −453.20]
cable #5	[531.5, −345, 441.5]	[539.36, −339.62, 430.26]	[538.07, −338.82, 430.34]
cable #6	[−531.5, −345, 441.5]	[−520.95, −341.69, 430.64]	[−522.64, −341.39, 430.22]
cable #7	[531.5, −345, 441.5]	[524.15, −347.9, 429.68]	[524.99, −347.57, 429.45]
cable #8	[531.5, 345, 441.5]	[536.57, 348.86, 430.48]	[534.50, 349.40, 429.55]
